# The effect of the leukoreduction filtration moment on the clinical outcome of transfused patients: A retrospective cohort study

**DOI:** 10.1016/j.clinsp.2025.100633

**Published:** 2025-04-08

**Authors:** Natasha Dejigov Monteiro da Silva, Lilia de Souza Nogueira, Youko Nukui, Cesar de Almeida-Neto

**Affiliations:** a Escola de Enfermagem da Universidade de São Paulo (EEUSP), São Paulo, SP, Brazil; b Hospital das Clínicas da Faculdade de Medicina da Universidade de São Paulo (HCFMUSP), São Paulo, SP, Brazil; c Fundação Pró-Sangue Hemocentro de São Paulo, São Paulo, SP, Brazil; d Disciplina de Ciências Médicas, Faculdade de Medicina da Universidade de São Paulo, São Paulo, Brazil

**Keywords:** Blood transfusion, Leukocyte reduction procedures, Transfusion reaction, Clinical evolution, Brazil

## Abstract

**Introduction:**

Leukoreduction is performed to decrease the occurrence of adverse effects of transfusion, and can be performed by pre-storage (bench or in-line) or post-storage filtration (bedside) moment. The authors verified the effect of the leukoreduction filtration moment of Red Blood Cell (RBC) and Platelet Concentrate (PC) on the occurrence of Adverse Transfusion Reactions (ATRs), the presence of Healthcare-Associated Infections (HAIs), Length of Hospital Stay (LOS), and hospital death.

**Methods:**

Retrospective cohort conducted at the *Hospital das Clínicas* of the Medicine Faculty of the University of São Paulo, and at the *Fundação Pró-Sangue Hemocentro* in São Paulo, Brazil. Adult patients, hospitalized for >24 hours, who received leukoreduced RBC and/or PC transfusion between 2017‒2020 were included. The generalized mixed effects model and the Wald test were applied in the analysis with a significance level of 5 %.

**Results:**

The authors evaluated 3668 patients who received 23,782 transfusions and we found no evidence of a leukoreduction filtration moment effect for ATR (*p* = 0.991) or HAI (*p* = 0.982), regardless of the transfused blood component. Meanwhile, the leukoreduction filtration moment had an effect (*p* < 0.001) on LOS, depending on the blood component transfused (*p* = 0.023), with pre-storage RBC filtration showing better performance, while in-line filtration stood out for PC. Both the leukoreduction filtration moment and the blood component (*p* = 0.041) influenced hospital death, with emphasis on the protective effect of bench RBC filtration and pre-storage PC filtration.

**Conclusion:**

The leukoreduction filtration moment associated with the blood component had an effect on the LOS and hospital death of patients undergoing transfusion.

## Highlights

• Leukoreduction decreases the adverse effects of blood component transfusion.
• Leukoreduction by pre-storage filtration reduced the length of hospital stay.
• Leukoreduction by pre-storage filtration reduced hospital deaths.
• No effect of leukoreduction filtration moment on adverse transfusion reaction.
• No effect of leukoreduction filtration moment on healthcare-associated infection.

## Introduction

Transfusion is a life-saving therapy, but has the potential to cause adverse effects such as immediate (within 24 hours of transfusion) or late (after 24 hours of transfusion) Adverse Transfusion Reaction (ATR), from mild to potentially fatal severity.^1^^,^^2^ Some ATRs occur because whole blood is collected, then processed into different blood components and subsequently stored. During storage, there is a release of inflammatory mediators resulting from the degradation of leukocytes, such as cytokines, interleukins (IL-1 and IL-6), and tumor necrosis factor. Furthermore, leukocyte antigens and metabolically active cells are capable of proliferating and producing immunological modifiers that affect the recipient during the transfusion. The recipient will concomitantly respond to these modifiers by producing their own immunological mediators that further influence the clinical response.^3^, ^4^, ^5^, ^6^

ATRs are estimated to range from three to five reactions for every thousand transfusions performed. They occur more frequently when Red Blood Cell (RBC) and Platelet Concentrate (PC) are transfused. Febrile Non-Hemolytic Transfusion Reaction (FNHTR) is the most frequent ATR.^7^, ^8^, ^9^ Furthermore, Transfusion-Related Immunomodulation (TRIM), meaning the effects of transfusions on the immune system, causes undesirable clinical events such as an increase in postoperative bacterial infections and recurrence of malignant diseases, reactivation of latent and asymptomatic infections, and increased morbidity and mortality of patients.^10^, ^11^, ^12^, ^13^, ^14^

Leukoreduction is a procedure performed with each blood component to reduce the occurrence of these abovementioned adverse transfusion events. Leukoreduced blood components must contain less than 5 × 10^6^ leukocytes per unit. Blood components, fresh frozen plasma, and cryoprecipitate do not require leukoreduction, as there is not a sufficient amount of residual leukocytes in these components that could be harmful to the recipient.^1^, ^2^, ^3^^,^^5^

Leukoreduction can be performed with filters, whether pre-storage (bench or in-line) or post-storage (bedside). Bench filtration is performed in the hemotherapy service laboratory within the first 48 hours after collecting the whole blood unit, and in-line filtration is between 2 and 24 hours after collection.^15^ The bedside filter is used during the transfusion.^16^ Leukoreduction by both pre-storage in-line and benchtop filtration avoids an accumulation of cytokines which are synthesized during cell storage, hemolysis of red cells, and filtration interruption caused by cell debris resulting from RBC storage. Post-storage filtration leukoreduction does not eliminate inflammatory mediators produced by the degradation of leukocytes which are responsible for the unfavorable clinical outcomes of recipients.^7^^,^^16^^,^^17^

Studies show the superiority of pre-storage leukoreduction filtration in relation to post-storage in reducing non-hemolytic transfusion reactions, infection, and death within 60 days of transfusion.^18^, ^19^, ^20^, ^21^, ^22^, ^23^ A recent systematic review showed that there are no studies in the literature that compare the effectiveness between the three filtration moments (bench, online and bedside) and outcomes related to the clinical evolution of patients such as ATR, infection, length of stay and hospital death, nor investigations carried out in Brazil.^24^ Given the limited availability of national and international studies comparing the effectiveness between the three moments of leukoreduction, this research aimed to investigate whether there are differences in the filtration moment (bench, in-line, or bedside) of RBC and PC on the occurrence of ATR, the presence of Healthcare-Associated Infection (HAI), the Length of Stay (LOS), and hospital death of transfused patients, irrespective of the underlying pathology.

## Material and methods

### Study design and location

The authors conducted a retrospective cohort study guided by the STrengthening the Reporting of Observational studies in Epidemiology (STROBE) tool, developed at the institutes of the Hospital das Clínicas of the Medicine Faculty of the University of São Paulo (*Hospital das Clínicas da Faculdade de Medicina da Universidade de São Paulo ‒ HCFMUSP*): State Cancer Institute of São Paulo (*Instituto do Câncer do Estado de São Paulo* ‒ *ICESP*), specialized in oncology; Heart Institute (*Instituto do Coração ‒ InCor*), specialized in cardiopneumology; Institute of Orthopedics and Traumatology (*Instituto de Ortopedia e Traumatologia ‒ IOT*), specialized in trauma and orthopedics; the Institute of Psychiatry (*Instituto de Psiquiatria – IPq*), which at the time performed neurosurgery procedures; and the Central Institute (*Instituto Central ‒ ICHC*), which serves several specialties and is a reference center for trauma care.

This study was approved by the *HCFMUSP* Ethics Committee (opinion n° 4,452,721,2,0000,0068), and because it was a retrospective collection with documentary analysis, the application of the Informed Consent Form to participants was waived.

### Participants

In the present study, the authors chose a convenience sample of patients aged 18 years or over who received a transfusion of leukoreduced allogeneic RBC and/or PC by pre- or post-storage filtration in the period from 01/01/2017 to 12/31/2020, and who remained hospitalized for at least 24 hours in one of the *HCFMUSP* institutes. Patients with a do-not-resuscitate order, brain dead and/or diagnosed with sepsis upon hospital admission were excluded from the sample. Patients who received leukoreduced RBC and/or PC filtration at different times (bench, in-line or bedside) during the same hospitalization were also not considered.

### Description of the filters used

Platelet collections by leukoreduced apheresis by in-line filtration were obtained using the Trima Accel® automatic blood collection system with the capacity to collect products with less than 5 × 10^6^ leukocytes per unit. The BIOR 01 PLUS BBS PF filter (for CH) and BIOP 10 Plus BBSS PF (for PC) were used in leukoreduction by bench filtration, both from Fresenius®, and with a residual leukocyte level lower than 2 × 10^5^ per unit. Leukoreduction by bedside filtration was performed using the Haemonetics® RC1VAE (Auto Prime) filter (for CH), with a residual capacity of less than 2 × 10^5^ leukocytes per unit, and the Haemonetics® PL3VAE filter (for PC) with platelet recovery greater than 90 % and residual leukocyte level less than 2 × 10^5^ per transfusion. Pre-storage leukoreduction was performed at the *Fundação Pró-Sangue do Hemocentro de São Paulo* (*FPS-HSP*), and post-storage at the time of transfusion in the patient admitted to one of the *HCFMUSP* institutes.

### Variables

The independent predictor variable of the study was the leukoreduction filtration moment, categorized as bench or in-line (pre-storage) or bedside (post-storage). The dependent outcome variables were the occurrence of immediate post-transfusion ATR (FNHTR, bacterial contamination, dyspnea or hypotension associated with transfusion and allergic reaction), presence of HAI within 72 hours after transfusion, LOS, and hospital death.

### Data source

Information regarding blood components and the presence of transfusions and previous reactions of patients was extracted from the database of requisitions and blood components dispensed by *FPS-HSP* during the study period. This information was cross-referenced with the electronic medical records database of the participating institutes, considering the study's eligibility criteria and searching for the variables of interest in the study. ATR occurrence was obtained from the electronic medical records of transfused patients, from the Health Surveillance Notification System (*Sistema de Notificação em Vigilância Sanitária ‒ NOTIVISA*) and from the Health Surveillance Center (*Centro de Vigilância Sanitária ‒ CVS*-4), which contains the mandatory record of the final destination of blood components prepared for transfusion in Brazil. Information on HAIs was extracted from the results of culture tests carried out within 72 hours after the transfusion and validated with the Hospital Infection Control Committees (*Comissões de Controle de Infecção Hospitalar ‒ CCIH*) of the *HCFMUSP* institutes. LOS and in-hospital death were identified from patients’ electronic medical records. The collected data were entered into the Microsoft Excel program® 2019.

### Statistical analysis

Categorical variables were expressed as absolute and relative frequencies. Numerical variables were presented as median and quartiles since the Shapiro-Wilk test showed a non-normal distribution. Considering that each patient may have received more than one transfusion during their hospital stay, the generalized mixed effects model for the binomial family was applied to verify the effect of the leukoreduction filtration moment for each dependent variable of the study controlled by the transfused blood component (RBC or PC). The set (cluster) of patients and observations of transfusions performed were considered When applying this model, the Wald test with Tukey's correction was applied to identify possible differences by blood component between bench, in-line, and bedside filtrations in relation to the analyzed outcome. A value of *p* < 0.05 was considered statistically significant. The data were analyzed using the *R* version 4.3.1 program.

## Results

A total of 151,737 RBC and PC were dispensed during the study period, with 23,782 leukoreduced units transfused in 3668 patients. Table 1 shows the characteristics of the patients in the sample.

**Table 1:** Demographic characteristics and blood type (*n* = 3668).

Variables	n (%)	Median (1st‒3rd quartile)
Sex		
Female	1519 (41.4)	
Male	2149 (58.6)	
Age		58,1 (44,8‒67,0)
Self-reported skin color^a^		
White	2252 (73.1)	
Brown	541 (17.5)	
Black	269 (8.7)	
Yellow	20 (0.7)	
Blood type^b^		
O	1745 (47.8)	
A	1340 (36.7)	
B	439 (12.0)	
AB	130 (3.5)	

a Missing (*n* = 586).

b Missing (*n* = 14).

Neoplasms (34.4 %), infectious and parasitic diseases (16.7 %), and diseases of the blood and hematopoietic organs (11.0 %) were the main diagnoses among the 4463 hospitalizations that occurred (3184 at *ICESP*; 1058 at *ICHC*; 180 at *InCor*; 37 at *IOT* and 4 at *IPq*). The transfusion characteristics are described in Table 2.

**Table 2:** Transfusion characteristics (*n* = 23,782).

Variables	n ( %)	Median (1st‒3rd quartile)
Previous tranfusion(s)		
Yes	21,999 (92.5)	
Ignored	1422 (6.0)	
No	361 (1.5)	
Previous ATR(s)		
Yes	19,602 (82.4)	
No	2668 (11.2)	
Ignored	1512 (6.4)	
Blood component		
Red blood cell	14,724 (61.9)	
Platelet concentrate	9058 (38.1)	
Number of transfusions per patient		2 (1‒5)
Leukoreduction filtration moment		
Bedside	22,980 (96.6)	
In-line	471 (2.0)	
Bench	331 (1.4)	

ATR, Adverse Transfusion Reaction.

The ATR incidence was 10.4 % (2470 cases). The most prevalent ATR symptoms were pruritus (56.2 %) and rash (13.5 %), and the most reported ATR diagnosis in *NOTIVISA* was FNHTR (55.6 %), followed by allergic reaction (34.3 %). A total of 514 HAIs were identified (2.2 %), with the bloodstream (*n* = 164; 31.9 %) and urine (*n* = 108; 21.0 %) being the predominant foci. The median LOS was 15.9 days (1st quartile 7.4; 3rd quartile 28.3), and 1382 patients (31.0 %) died.

**Fig. 1:**
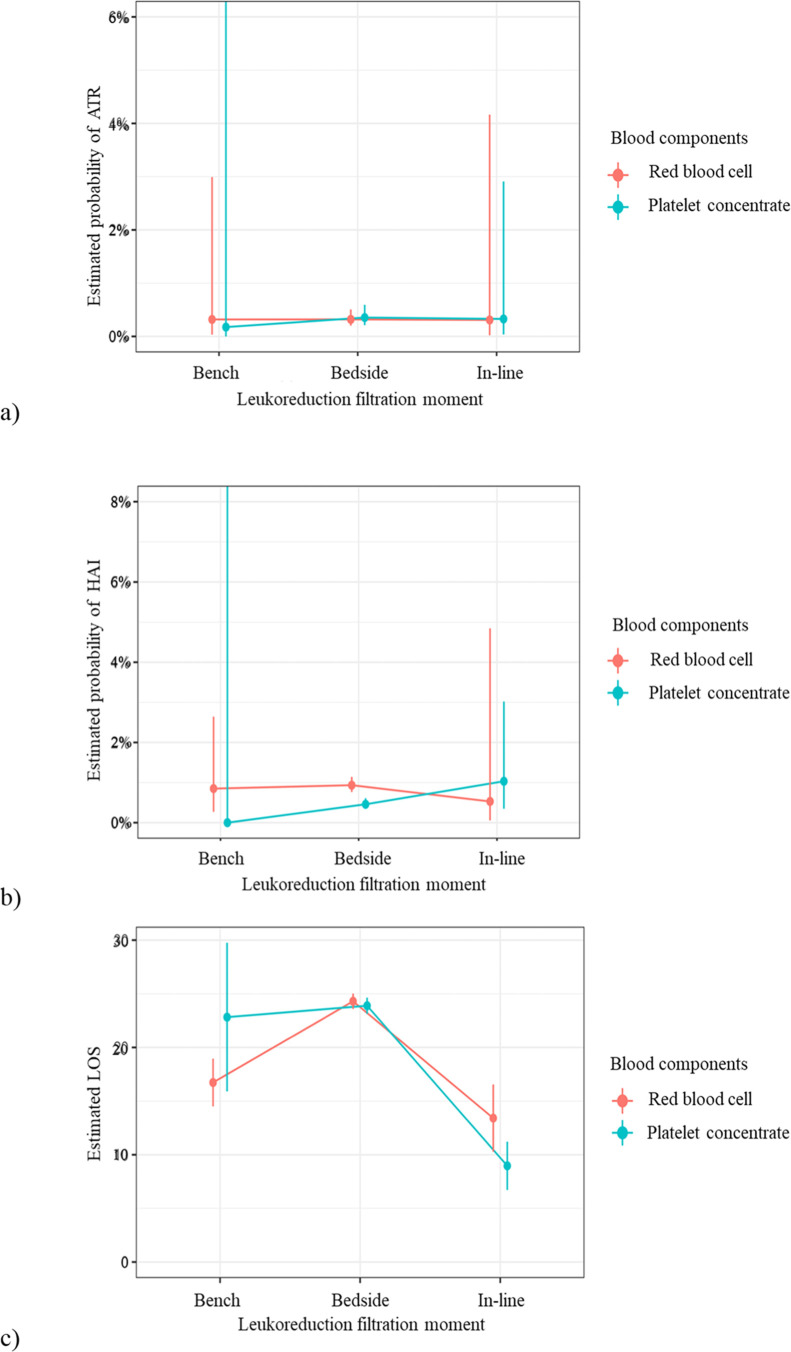
Graphical representation of estimates of ATR, HAIs, LOS and hospital death according to the leukoreduction filtration moment and blood component type.

There is no statistically significant evidence that the leukoreduction filtration moment has an effect on ATR occurrence (*p* = 0.991), regardless of the blood component transfused (*p* = 0.993) (Table 3 and Fig. 1a).

**Table 3:** Effect of the leukoreduction filtration moment on ATR occurrence.

	χ^2^	df	p^a^
**(Intercept)**	30.93	1	<0.001
**Leukoreduction filtration moment**	0.02	2	0.991
**Blood component**	0.01	1	0.944
**Leukoreduction filtration moment: blood component**	0.017	2	0.993

ATR, Adverse Transfusion Reaction; χ^2^, Chi-squared; df, degrees of freedom.

a Generalized mixed effects model for binomial family.

For the analysis of the presence of HAIs, the findings showed that the effect of the leukoreduction filtration moment on the outcome was similar (*p* = 0.982), regardless of the blood component administered (*p* = 0.558) (Table 4, Fig. 1b).

**Table 4:** Effect of the leukoreduction filtration moment on the presence of HAIs.

	χ^2^	df	p^a^
**(Intercept)**	0.00	1	0.967
**Leukoreduction filtration moment**	0.04	2	0.982
**Blood component**	0.00	1	0.990
**Leukoreduction filtration moment: blood component**	1.17	2	0.558

HAIs, Hospital-Associated Infections; χ^2^, Chi-Squared; df, degrees of freedom.

a Generalized mixed effects model for binomial family.

The data in Table 5 and Fig. 1c show that the leukoreduction filtration moment had an effect (*p* < 0.001) on the patients’ LOS, depending on the blood component transfused (*p* = 0.023). For RBC transfusion, patients who received leukoreduced through bedside filtration had a higher estimated mean LOS (24.3 days) than the pre-storage group for both bench (16.7 days) and in-line (13.4 days). For PC transfusion, the group that received in-line leukoreduction filtration had a lower estimated mean LOS (9.0 days) than those who received it bedside (23.9 days) or bench (22.8 days).

**Table 5:** Effect of leukoreduction filtration moment on LOS.

	χ^2^	df	p^a^
**(Intercept)**	589.74	1	<0.001
**Leukoreduction filtration moment**	167.99	2	<0.001
**Blood component**	0.09	1	0.764
**Leukoreduction filtration moment: blood component**	7.58	2	0.023

LOS, Length of Hospital Stay; χ^2^, Chi-Squared; df, degrees of freedom.

a Generalized mixed effects model for binomial family.

The model also showed that both the leukoreduction filtration moment and the blood component (*p* = 0.041) had an influence on the hospital death outcome (Table 6). Patients who received leukoreduced RBC through bench filtration had a lower estimated probability of death in the hospital (3.2 %) than in-line (25.9 %) or bedside (24.6 %). The estimated probability of hospital death for PC was significantly lower for in-line filtration (4.0 %) compared to bedside (27.4 %). A low estimated probability of death (0.5 %) was also observed among patients who received PC leukoreduction bench filtration, and a possible difference between this type of filter and the bedside was not identified due to the low number of cases in this group (Fig. 1d).

**Table 6:** Effect of leukoreduction filtration moment hospital death occurrence.

	χ^2^	df	p^a^
**(Intercept)**	21.92	1	<0.001
**Leukoreduction filtration moment**	9.79	2	0.007
**Blood component**	1.60	1	0.206
**Leukoreduction filtration moment: blood component**	6.38	2	0.041

χ^2^, Chi-Squared; df, degrees of freedom.

a Generalized mixed effects model for binomial family.

The summary of the results found in the research is presented in Table 7.

**Table 7:** Summary of the research findings.

Blood component	Leukoreduction filtration moment	Effect on clinical outcome			
		ATR	HAIs	LOS	Hospital death
RBC	In-line	No	No	Yes	No
	Bench	No	No	Yes	No
	Bedside	No	No	No	Yes
PC	In-line	No	No	Yes	No
	Bench	No	No	No	No
	Bedside	No	No	No	Yes

ATR, Adverse Transfusion Reaction; HAIs, Hospital-Associated Infections; LOS, Length of Hospital Stay.

## Discussion

In the present study, the leukoreduction filtration moment associated with the blood component had an effect on the LOS and hospital death of the patients in the sample.

Patients who received RBC transfusion, whether leukoreduction bench or in-line filtration, spent less time in the hospital. In-line filtration showed better performance for those receiving PC transfusion than bench or bedside filtration. A single study was found in the literature comparing the length of stay in the Intensive Care Unit (ICU) between three groups: patients who received blood components without buffy-coat, blood components leukoreduced by post-storage filtration, and blood components leukoreduced by pre-storage filtration. The researchers identified that the group transfused with a blood component without a buffy coat remained hospitalized in the ICU for longer (3.5 days) than those transfused with a leukoreduced blood component pre (3.0 days) or post-storage (3.2 days).^20^ These findings reinforce the benefits of leukoreduction, as it enables an increase in bed turnover and enhances hospital performance.

The mortality rate found in this study was higher in the group of patients who received leukoreduced blood components through bedside filtration. The benefits of leukoreduction on the survival of transfused patients are inconclusive in the medical literature. Some research has shown that the leukoreduction of allogeneic blood components was associated with a reduction in mortality in patients undergoing massive transfusion,^13^ heart valve replacement,^25^ or acute lung injury.^26^ Furthermore, implementation of the Canadian Universal Leukoreduction Program for RBC showed that patients who received leukoreduced transfusions had an approximately 1 % lower mortality rate than patients who received non-leukoreduced transfusions.^27^ On the other hand, some studies have not identified the benefits of using leukoreduction in reducing mortality,^14^^,^^28^ nor the effect of implementing universal leukoreduction on the survival of transfused trauma patients.^29^ It is well-established that the number of transfusions performed impacts hospital mortality^30^ However, the use of pre-storage leukoreduction has the potential to improve patient survival rates.

The present study identified that both the leukoreduction filtration moment and the blood component had an influence on the occurrence of death in the sample, and leukoreduction bench filtration was less likely to be associated with the clinical outcome for RBC, while the highlights for PC were in-line and bench filtration. Research showed that mortality within 60 days after cardiac surgery was lower in groups of patients who received leukoreduced transfusions (pre- or post-storage) compared to those who received only blood components without buffy-coat.^20^ It should be noted that no studies were identified that compared the three leukoreduction filtration moments in relation to the clinical evolution of transfused patients (including ATR, HAIs, LOS and hospital death), making it impossible to compare the results of this study in more depth with the scientific literature.

This study showed a higher ATR incidence rate than that found in the literature^31^^,^^32^ and the reference to the *Agence Régionale de Santé Île-de-France* used by the National Health Surveillance Agency (*Agência Nacional de Vigilância Sanitária - Anvisa*) of Brazil.^7^^,^^33^ It is worth highlighting that this high rate may be related to campaigns carried out by both *HCFMUSP* and *FPS-HSP* with the aim of raising awareness among professionals about the importance of reporting adverse events and reducing underreporting of ATR, and the conditions reported in different studies, such as the largest challenge of hemovigilance.^34^^,^^35^ Furthermore, some adverse drug reactions may have been confused with ATR, especially in patients who were polymedicated or who received chemotherapy. Considering only the notifications sent to *Anvisa*, this study found that the main diagnosis was FNHTR, followed by an allergic reaction, similar to what was demonstrated in some studies that evaluated the ATR frequency.^34^, ^35^, ^36^ However, it is worth noting that the pathophysiology regarding the allergic reaction is related to the presence of anti-IgE antibodies in the receptor, or in rare cases, to IgA deficiency with the formation of anti-IgA5 antibodies, with no indication for the use of leukoreduction for preventing this type of reaction.

In the present study, no effect of the leukoreduction filtration moment on ATR occurrence was identified, regardless of the blood component transfused. This topic is controversial in the literature. There are studies that identified that there was no significant difference between the leukoreduction filtration moments and ATR occurrence after PC^37^ or RBC transfusion^38^; however, it is worth highlighting that these studies^37^^,^^38^ evaluated different usage periods for each type of filtration. On the other hand, an American study that also evaluated different leukoreduction filtration process periods identified that the FNHTR incidence was significantly lower in the pre-storage leukoreduction group for both RBC and PC.^23^ A similar finding was verified in a study conducted in 15 American hospitals in Pittsburgh which analyzed the presence of FNHTR after PC transfusion and identified lower reaction rates among those who received the pre-storage filtered blood component (0.07 %) compared to post-storage (0.16 %).^21^ The FNHTR frequency in the first four hours after transfusion in a hospital in Taiwan was lower in the group of patients who received RBC pre-storage leukoreduced filtration.^22^ A study published in 2024 suggests that pre-storage leukoreduction of platelet apheresis is the ideal choice for supplying this blood component.^18^ These contradictory findings may be associated with the characteristics of the patients included in the studies (oncology patients, post-transplant patients, immunocompromised patients, etc.), the different sample sizes and the scope of the data analyzed.

The presence of HAIs 72 hours after the transfusion was only found in 2.16 % of the sample in this study. Research results show that patients who receive leukoreduced blood components have a lower infection risk than those who receive non-leukoreduced transfusions,^39^^,^^40^ which may explain the low HAI incidence in this study. No relationship was identified between the leukoreduction filtration moment and the presence of HAIs 72 hours after transfusion. The results in the literature are also contradictory. In patients undergoing colorectal resection surgery, pre-storage leukoreduction filtration had a positive effect in preventing infectious complications, especially bloodstream infection, pneumonia, and urinary tract infection.^19^ It should be noted that the patient follow-up period was not described in this study,^19^ making it impossible to say whether the infections occurred during hospital stay. On the other hand, Dutch researchers evaluated the occurrence of infection within 60 days after cardiac surgery and identified that the frequency of infections was similar among patients who received PC, regardless of the leukoreduction filtration moment.^20^

Some limitations of this study must be addressed. Bedside leukoreduction filtration was considerably the most used in patients in this study, which is consistent with the practice adopted in Brazilian public hospitals and different from that found in countries in Europe and North America, where universal leukoreduction is already well established and post-storage filtration is no longer used. The study was conducted in a hospital complex which, despite being composed of different institutes, follows the same recommendations and procedures in a corporate manner, which must be considered when generalizing the results. It is also known that some factors not evaluated in this study, which included highly complex patients, have the potential to influence the results or be considered confounding factors, such as the use of premedication to minimize some unwanted transfusion symptoms or even the fact that an ATR is considered a reaction associated with medications. Furthermore, as this was a retrospective study, data gaps in the medical records and the use of a passive ATR notification system made it difficult to retrieve some information.

Finally, it is worth highlighting that modern transfusion medicine offers numerous tools to improve the efficacy and safety of transfusion, including universal leukoreduction. Although its implementation requires substantial financial and infrastructure resources, it should be considered to improve the quality and safety of transfusion therapy for vulnerable patients. Implementing leukoreduction through pre-storage filtration can be advantageous, or at the very least, contribute to the hospital's performance by increasing bed turnover and reducing the mortality rate, as the study suggests.

In conclusion, these results showed that both the leukoreduction filtration moment and the blood component transfused had an effect on some clinical outcomes evaluated in the study. For LOS, pre-storage filtration performed best for red blood cells, and in-line filtration stood out for PC. In hospital deaths, bench filtration had a protective effect on red blood cells and pre-storage filtration (bench or in-line) for PC. However, there was no effect of leukoreduction filtration moment in the occurrence of RTA and IRAS after transfusion, which demonstrates that patient characteristics may have influenced these outcomes. The present results found a relevant impact on management indicators, especially the increase in the index bed turnover and reduction in the institutional mortality rate.

While cost assessment was not the primary focus of this study, optimizing the allocation of healthcare resources remains essential. Shifting resources from the acquisition of post-storage filters to the implementation of pre-storage leukoreduction offers a potentially more cost-effective alternative. However, further research is needed to assess the broader impact of this approach on healthcare service costs.

## Funding

This work was financed in part by the Coordenação de Aperfeiçoamento de Pessoal de Nível Superior ‒ Brasil (CAPES) ‒ Finance Code 001.

## Conflicts of interest

The authors declare no conflict of interest.
